# Supplementary material for: Are carbon emissions trading and green financial instruments synergistic? -Comprehensive quantitative research based on content analysis
Source: PLoS One. 2024 Mar 7;19(3):e0298601. doi: 10.1371/journal.pone.0298601 (PMC10919601; doi:10.1371/journal.pone.0298601)
Supplement: S1 Data — (ZIP) [file pone.0298601.s001.zip › Data/地级市Z_F工作报告.zip!/║■─╧/╔█╤⌠╩╨/└┤╫╘╣1⁄2╓┌║┼í╛┬φ┐╦╩2╛▌═°í┐_2012.docx]

各位代表：

　　现在，我代表市人民政府，向大会报告工作，请予审查。并请各位政协委员和列席人员提出意见。

　　

　　一、2011年工作回顾

　　2011年是"十二五"规划的开局之年。全市上下在市委的正确领导下，按照"四化两型"战略部署，坚持科学发展，加快后发赶超，实现富民强市，圆满完成了市十四届人大四次会议确定的目标任务。

　　1、推进结构调整，经济实力持续增强。地区生产总值达到907.2亿元，增长13.2%，增速比全省高0.4个百分点。产业结构进一步优化，三次产业比例由上年的23.8:38.1:38.1调整为23.4:40.2:36.4。财政收入达到66.3亿元，增长27.8%，其中市本级财政收入达到17.4亿元，增长28.5%。金融机构存款余额达到1116.2亿元、贷款余额达到425.4亿元，分别增长17.7%、22.1%。保费收入达到34.3亿元，增长13.9%。工业经济快速发展。规模工业增加值达到294.1亿元，增长22%，增速比全省高2.5个百分点、居全省第一位。建材、食品、机械、冶金、能源、医药化工、造纸、纺织八大优势产业增势强劲，其中建材、食品和机械三大产业产值突破百亿元，分别达到154亿元、150亿元和130亿元。"能源城、汽车城和美酒城"初具雏形。宝庆电厂一期工程竣工发电。三一湖汽产业园一期工程部分竣工投产。湘窖酒业二期工程1万吨浓香型酿酒车间正式出酒。邵阳纺机退城入园整体搬迁抓紧进行，九兴鞋业落户宝庆科技工业园，南山风电一期工程竣工发电，立得皮革、湘中制药、玉新药业等企业新基地基本建成。企业改制平稳推进。全市新增规模工业企业55家，产值过亿元企业达到330家。园区完成工业总产值520亿元，增长45%。农业经济稳步增长。完成农林牧渔增加值达到217.6亿元，增长3.7 %。粮食播种面积795.6万亩，总产310.8万吨，连续八年增产丰收。袁隆平院士的超级杂交水稻在隆回试验，创造了亩产926.6公斤的记录。桔橙、果蔬、烟叶、药材和生猪、肉牛、山羊、水产等种植业和养殖业都有较大发展。农产品加工企业发展到4017家，其中规模企业312家，新增16家。农民专业合作组织发展到1510个。有机食品、绿色食品和无公害农产品认证产品达到248个。造林27万亩，森林覆盖率提高到57.6%。建成户用沼气池11667口、大中型沼气工程7处。推广各类农机具6万台（套）。新农村办点示范和连片建设成效显著，打造示范村209个、示范片44个。商贸旅游活力增强。实现社会消费品零售总额328.5亿元，增长18%，增速比全省高0.2个百分点、居全省第二位。"万村千乡"市场工程和"家电下乡"活动扎实推进，销售家电下乡产品60万台，家电以旧换新16.5万台。加强市场监管，物价基本稳定。邵东县廉桥药材市场被评为全国物流先进单位。全年引进外资达到1.4亿美元、增长27.9%，利用内资达到422.9亿元、增长16.4%，实现进出口总额达到3.9亿美元、增长32.8%。新批境外投资企业33家，为年计划的254%，新增境外投资企业数和境外投资企业总数均居全省首位。隆回荣获中国最佳民俗风情旅游名县称号，洞口县 溪被批准为国家级森林公园，湘窖酒业被授予国家3A景区。武冈市列为省级历史文化名城，新宁县建成湖南旅游强县。全年实现旅游收入80亿元，增长45.6%。第三产业增加值达到327亿元，增长13.1%，增速比全省高1.1个百分点。

　　2、强化项目建设，基础条件不断完善。完成固定资产投资562.1亿元，增长35.2%，增速比全省高7.1个百分点、居全省第五位。实施重点工程124个，其中新开工5000万元以上的投资项目62个。交通建设方面。洞新、娄新、邵安、邵坪、包茂5条高速公路建设进度加快。国省干线公路改造完成投资11亿元，完成项目8个、154公里，为省定目标的136%。建成农村公路1444.6公里，其中县乡道352.2公里、通畅工程1092.4公里，分别为省定目标的167.7%、109%。建成了一批县、乡、村客运车站。娄邵铁路扩改工程和沪昆高速铁路新建工程进展顺利，邵阳武冈机场新建工程和邵东机场改建工程前期工作步伐加快。水利建设方面。44座小一型水库除险加固主体工程全部完成，89座小一型与89座小二型病险水库治理正在组织施工。7个中小河流治理项目全面启动。衡邵干旱走廊综合治理前期工作抓紧进行。新建农村安全饮水工程164处，解决38.3万人饮水不安全问题。能源建设方面。新一轮农网改造启动，完成电网投资10亿元，为"十一五"期间年均水平的四倍。新建扩建变电站28座。雪峰山20万千瓦风力发电项目签约。

　　3、推进扩容提质，城镇面貌明显改观。城乡规划更加完善。城乡一体化建设进入新阶段，村庄布局规划全面启动，建制镇总体规划编制完成，县城详细规划覆盖率提高到76%。中心城市变化较大。西湖南路、东大路、东塔路、双拥路、敏州中路、建设南路延伸段和魏源广场等实现了高标准改造，学院路扩改工程强力推进，大祥路、财神路、桂花路、雪峰南路、新华路南段竣工通车，西苑生态公园和爱莲池公园启动主广场建设，时代公园和蔡锷广场即将建成。行政中心建设前期工程扎实推进。管输天然气入邵工程和人防1206工程动工建设，人防1505工程已经建成。11个污水处理项目负荷率75%以上。生活垃圾无害化处理率90%以上。中心城区新增建设用地3860亩，建成区面积达到57平方公里。城市管理继续加强。开展"五城同创"，整治市容环卫秩序，收到较好效果。主干道亮化率100%，重要临街建筑全部安装霓虹灯，10层以上高层建筑和标志性建筑大都装配了发光二极管灯，人均公共绿地面积达到5.2平方米。县城和中心城镇建设步伐不断加快。全市城镇化率达到34.1%。

　　4、扩大公共投入，社会事业协调发展。各类教育协调并进。普九成果巩固提高，义务教育阶段三率全部达标。职业教育发展迅速，民办教育繁荣兴旺。大中专院校建设获得新发展，邵阳学院申硕成功。完成学校建设项目712个，办学条件逐步改善，市区祥凤学校即将竣工。科技创新步伐加快。产学研结合日益紧密，130多家企业与高校、科研院所开展技术合作。3家企业纳入省级工程技术研究中心建设，67个项目列入国家和省级科技计划。专利授权550件，"国家知识产权试点城市"通过国家验收。高新技术产业产值达到230亿元，增长33%。文化事业不断进步。"三馆一站"免费开放正式启动。乡镇文化站101个建设项目全部完成。农家书屋达到3935个，覆盖全市行政村的71%。群众文化活动丰富多彩，400多个基层群众文娱团队成为社区文化亮点。城步吊龙、武冈丝弦入选第三批国家级非物质文化遗产名录。文化体制改革加速推进。文化市场整治力度加大，文化监管工作被评为全国先进。全市广播和电视混合人口覆盖率分别达到78.2%和92.2%。中国君子文化产业园项目启动。全市文化产业收入达到26亿多元，逐步成为新的经济增长点。公共卫生服务加强。改造了一批县乡医院。基本药物制度全面覆盖。重大疫病防控工作扎实开展。新农合"普惠安全"工程和"双十"便民惠民行动深得群众好评。体育运动蓬勃发展。成功承办了国家和省里赛事5项，参加国家级比赛获3金6银7铜的优异成绩。人口资源环境工作水平提高。人口计生工作继续保持全省先进。符合政策生育率达到86.7%，提高4.5个百分点；出生人口性别比117.2，下降近1个百分点。耕地保护基本国策和资源节约优先战略进一步落实，新增耕地9965亩，连续十年实现耕地占补平衡。维护矿产资源开发秩序工作连续四年全省先进。节能减排成效明显，城乡环境质量持续改善。整治污染力度进一步加大，取缔关闭高污染和高能耗企业63家，万元规模工业增加值能耗降低11%，年度节能减排任务全面完成。

　　5、重视民生民利，群众生活逐步改善。城镇居民人均可支配收入达到13578元，增长15.1%，增速比全省高1.3个百分点、居全省第一位，增速为我市1995年以来连续16年之最。农民人均纯收入达到4373元，增长16.3%，增速为我市1997年以来连续14年之最。惠农政策全面落实，发放各类惠农补贴10亿元，落实"一事一议"财政奖补1.8亿元。突出民生价格监管，发放低收入困难群体价格临时补贴2365万元。省定和市定实事工程全面完成或超额完成。就业服务不断加强。新增城镇就业4.5万人，失业人员再就业2.9万人，新增农村劳动力转移就业6万人，零就业家庭动态援助达到100%。小微企业劳动合同签订率达90.1%，实行工资集体协商的企业达到1340家。建成了一批大中专毕业生创业孵化基地、创业基地和见习基地。社会保障更加完善。新增企业基本养老、医疗、失业、工伤、生育保险参保人数20.5万人，新增农村养老190万人，完成各类社保基金征缴25.7亿元，发放社保基金38亿元，受益群众98.8万人。新型农村合作医疗覆盖603.9万人，参合率97.4%。5个县市纳入第三批新农保试点，城镇居民养老保险、失地农民社会保险试点启动。城乡低保对象分别达到13.6万人和33.7万人。新建和改扩建敬老院15所，新增集中供养五保对象773人。完成农村危房、国有林场危旧房改造14023户、3819户。救助城乡贫困群众30.7万人次，发放救助金5462.4万元。大山岭陵园竣工使用，殡葬改革初显成效。烈士陵园已经建成。征地拆迁补偿安置制度改革稳步推行，保障性住房建设超额完成任务，新增廉租房8657套、43.2万平方米，新增公租房1138套、6.8万平方米，改造城市和国有工矿棚户区63.2万平方米，发放廉租住房补贴3304万元。住房公积金覆盖23.9万人，发放个人住房贷款21亿元，8.6万人使用住房公积金改善了住房条件。扶贫开发扎实推进。争取财政扶贫资金1.6亿多元、贴息贷款资金5亿多元，直接帮扶9.6万人脱贫致富。8个县市列入国家武陵山集中连片扶贫攻坚重点县，邵东县和三区列为国家武陵山集中连片扶贫攻坚重点县政策比照县区。全省唯一的农民培训基地落户我市。农业综合开发完成投资5.8亿元，建设土地治理项目区16个，实施产业化经营项目28个。社会大局和谐稳定。大力开展"清网行动"，网上逃犯"清网率"全省第一。坚持从严治邵，依法打击各种违法犯罪活动，社会治安秩序持续稳定。努力化解矛盾纠纷，信访工作连续七年被评为全省先进。群众饮食用药安全较好维护。重大动物疫病有效防控。突发应急事件妥善处置。加强安全监管，全市安全生产形势总体平稳。

　　6、改进管理服务，政府建设得到加强。推进政府职能转变和管理创新。认真办理行政复议案件，清理和取消行政许可项目321项，减少行政事业性收费23项，修改和废止规范性文件63件。自觉接受人大的法律监督、工作监督和政协的民主监督，注意听取各民主党派、工商联、无党派人士和各人民团体的意见，办理人大代表建议215件和政协委员提案345件，满意率均达99%。深化政务公开，发展环境优化。电子政务和电子监察系统建成运行。"五五"普法任务圆满完成。严格执行《廉政准则》，加强行政监察和审计监督，政风建设取得新成效。

　　与此同时，统计、工商、质监、市志、档案、贸促、移民、人事编制、民族宗教、外事侨务、邮政通讯、气象水文、防震减灾、无线电管理等工作取得新的成绩，妇女、未成年人、老年人、残疾人权益得到较好保障，工会、共青团、妇联、残联、文联、科协、社科联、红十字会等群团事业得到长足发展，国家安全、国防动员、人民防空、拥军优属、民兵预备役工作不断进步。

　　各位代表，上述成绩的取得，离不开省委、省政府和市委的正确领导，离不开市人大、市政协和各民主党派、工商联、无党派人士、人民团体以及社会各界的监督支持，离不开800万邵阳各族人民的团结奋斗。在此，我代表市人民政府向全市广大工人、农民、干部、知识分子和企业家，向离退休老领导、老同志，向各民主党派、工商联、无党派人士、各人民团体和社会各界人士，向驻邵解放军、武警官兵和政法干警，向所有参与、支持与关心邵阳发展的投资者、建设者和朋友们，表示衷心的感谢和崇高的敬意！

　　各位代表，在肯定成绩的同时，我们也清醒地看到，我市经济社会发展还存在一些突出矛盾和问题，政府工作还有不少需要改进的地方。一是发展不快的问题仍然突出。邵阳在全省"人口总量居前、发展水平居后"的局面没有根本改变。尽管我市地区生产总值达到907.2亿元，但仍处于全省中等偏下位置，不足全省总量的5%，人均地区生产总值仅为全省平均水平的41%。二是财政困难的问题仍然突出。骨干财源企业不多，人均财政收入不足全省的四分之一，财政收支矛盾没有明显缓解。三是环境不优的问题仍然突出。少数单位和政府工作人员执行能力、服务水平有待进一步提高。对这些问题，我们将在今后的工作中，采取有力措施切实加以解决。

　　二、2012年主要任务

　　2012年是实施"十二五"规划承上启下的重要一年。这一年，尽管我市将面临更加复杂的发展形势，但多年改革发展积累的有利因素日益增多，加快发展的内生动力日益增强。特别是我市又整体纳入国家武陵山区域发展范围，同时还进入全国老工业基地调整改造规划城市，加快赶超发展的政策优势更加明显。只要我们科学把握形势，在挑战和机遇面前争取主动，就一定能推动邵阳新一轮更高水平和更好质量的发展。

　　工作总体要求是：全面落实科学发展观，按照"四化两型"要求，认真贯彻省、市第十次党代会和中央、省、市经济工作会议精神，紧紧抓住科学发展这个主题和加快转变经济发展方式这条主线，高举加快发展旗帜，坚持稳中快进方针，推动经济社会发展再上新台阶，努力创造800万邵阳人民的美好生活。

　　主要预期目标是：地区生产总值增长12%；规模工业增加值增长20%；全社会固定资产投资增长30%；社会消费品零售总额增长16%；财政收入增长16%；城镇居民人均可支配收入增长10%以上；农村居民人均纯收入增长11%以上；居民消费价格涨幅控制在5%以内；城镇登记失业率控制在4.5%以内；人口自然增长率控制在7.5‰以内；万元生产总值能耗下降3.4%；主要污染物排放总量控制在省政府下达的目标之内。

　　1、努力推动全市经济转型发展

　　加快推进新型工业，促进产业转型升级。围绕形成以年产值百亿元企业为龙头、五十亿元企业为重点、十亿元企业为骨干、亿元企业为群体的"1511"新型工业化体系，壮大优势产业，充分发挥新型工业化第一推动力作用。加快现有产业转型升级。以现代科研技术更新现有工艺流程，改造提升冶金、造纸、食品、纺织等传统产业，实现产品升级换代、产业迅速扩张；依托矿产、竹木、水电、风力等资源优势，扶优扶强建材、能源等特色产业，实现开发能力提升、产能迅速扩大；对接国家和省里有关新兴产业政策，支持帮扶电子、新材料、装备制造、生物医药等新兴产业，实现产业发展壮大、抢占战略高地。用足用活老工业基地改造政策，强力推进企业改制，抓好已改制企业扫尾验收，力争三年内完成市本级国企改制任务。大力扶持骨干企业发展。争取三一湖汽邵阳产业园项目一期工程、宝庆电厂二号机组、宝兴科肥合成氨、湘中制药丙戊酸系列产品异地扩建一期工程、玉新药业甾体医药中间体系列产品产业化项目、立得皮革650万张猪牛皮生产线竣工投产。南山风力发电、广信纸业扩改、洞口华菱矿业、隆回雨润食品、湘窖酒业二期、李文食品二期、九兴鞋业产业园、万事达退城入园、豫湘工贸生物繁育体系、恒天九五邵阳纺机新产业园、信多利稀土发光材料技术改造、维克液压低噪高压柱塞泵技改扩建等重点项目取得实质性进展，力争年内建成投产。加强调煤保电、银企合作等工作，强化生产要素保障供应，确保全年新增工业产值200亿元以上。发挥工业园区聚合作用。以工业园区为基点，以交通路网为联轴，注重产业特色，加大投入力度，在中心城区建设宝庆科技工业园邵阳大道沿线装备制造和高新技术产业带、邵阳经济开发区食品及纺织产业带，在县市建设"邵阳县-邵东-新邵"东部现代制造工业产业带、"隆回-洞口-武冈"中部食品混合产业带、"新宁-城步-绥宁"西线农产品开发产业带。启动本地产业配套工作，发展行业协会，逐步形成本地产品配套协作体系。抓好宝庆科技工业园和邵阳经济开发区"两化融合"试验区试点，整合资源争创国家级经济技术开发区。

　　加快建设新型城镇，促进城乡统筹发展。按照"东部城市群、西部生态圈"的思路，全面完成中心城区总体规划修编，加速编制东部城市群和城区交通体系规划，争取在省级层面启动东部城市群建设试点，带动城乡一体发展。完善城市功能。突出新区路网、旧城改造、社区配套，重点抓好"一路两桥三公园四工程"和资江、邵水风光带及高速公路两个出口建设，完成学院路及行政中心周边路网建设扩改工程。努力建成湖口井路、邵水东路南段、邵水西路南段、资江南路延伸段。开展新一轮小街小巷提质改造。加大北塔公园、东塔公园建设力度。推进资江南路步行街延伸段建设。抓好江北污水处理厂一期工程，做好洋溪桥污水处理厂管网配套和市污泥处置中心两项目前期工作。完成管输天然气入邵管道铺设，建好接收门站，年底实现供气。在确保质量的前提下，加快1206人防工程建设。建成市机动车驾驶员考试中心。提升城市品位。继续开展"五城同创"活动，开展以交通秩序、环境卫生为重点的城市综合管理，提高城镇文明程度。完善公用设施配套，建设一批公厕、垃圾中转站、农贸市场和社会停车场，改造或增设一批果皮箱、垃圾围和交通标志标识。推进城乡清洁行动，县城以上城镇生活垃圾无害化处理率保持90%以上。继续抓好资江、邵水中心城区段清污。启动"两江两岸"亮化工程，加强小街小巷灯化美化，规范设置主干道路两旁门店招牌和灯饰广告。强力推进拆违控违工作。提高城镇物业管理实施率。加快城乡一体发展。发挥中心城区引领作用，突出县城和重点城镇基础设施与中心城区的联结完善工作。加快城镇供电、供气、给排水等公用设施向农村延伸。加强乡村建设规划管理。

　　加快发展现代农业，促进农民持续增收。依靠科技创新驱动，大力推进农业现代化，建设社会主义新农村。突出发展现代农业。实施千亿斤粮食产能工程建设，确保粮播面积880万亩、总产340万吨以上。积极发展生猪、果蔬、桔橙、药材、油茶、楠竹、烟叶等特色产业，打造一批高标准生产基地，培育一批农产品精深加工骨干企业。全市经济作物总面积稳定在370万亩以上，突出抓好洞口雪峰蜜桔、新宁崀山脐橙和隆回金银花的扩面提质。大力发展生猪、肉牛、山羊生产。坚决实现烟叶生产30万担的奋斗目标。不断发展壮大农业、林业产业化龙头企业。积极发展农村第三产业，抓好全国休闲农业与乡村旅游示范县和示范点建设，打造全省休闲农业强市。推广各类农机具，争取每个县市区建好1个大型农机化示范基地。拓展农民增收渠道。加强农民转移就业培训，完成就业培训9.5万人，转移农村剩余劳动力130万人。引导企农合作，发展壮大农民专业合作组织，增强城镇对农村人口转移的吸纳能力，拓宽农民就业创业领域。全面落实惠农减负政策。严格涉农收费管理，切实减轻农民负担。抓好农业综合开发。投入开发资金6.5亿元，建设土地治理项目区16个，实施产业化经营项目21个。加大扶持力度，促进民族地区和高寒山区发展。推进新农村建设。按照"办点示范、连片推进、逐步扩面、整体提高"思路，推进第三轮市级重点示范村建设，打造示范村219个、示范片46个、示范镇14个。

　　加快基础建设步伐，促进发展后劲增强。改善交通条件。加快洞新、娄新、邵安、邵坪、包茂5条高速公路建设步伐，完成衡邵高速公路延长线建设，争取武靖、白新、洞溆、安北、武永高速公路开工建设。新改建邵新、隆武和新邵武汤、隆回六金、洞口洞山、绥宁长河、城步边南、邵东八老、邵东黄陶、邵阳五罗等干线公路17条，共计726公里。建成农村公路510公里。推进娄邵铁路扩能改造和沪昆高速铁路建设，争取怀邵衡铁路开工建设。力争武冈机场年底动工，加快邵东机场改建工程的前期工作。抓紧双筱航道整治工程设计，振兴邵阳水运。加强水利建设。编制完善邵阳水利中长期规划、资江流域水利综合规划，加快衡邵干旱走廊综合治理项目前期工作，启动木瓜山水源工程和犬木塘水库工程项目。汛期前完成89座小一型水库除险加固主体工程，年底完成89座小二型病险水库治理任务，争取3座中型水库、133座小二型水库、11座大型水闸的除险加固工程开工建设。建设农村饮水安全工程，解决30万农村人口安全饮水问题。启动控制性防洪枢纽工程建设，开展灌区续建配套与节水改造。加强河道整治，严厉打击乱采滥挖等非法采砂行为。强化能源保障。推进南山风电、雪峰山风电、白马山风电、大云山风电、龙山风电、宝顶山风电以及隆回、邵阳县生物质能发电项目建设，抓紧农网和城网改造，新建220千伏输变电工程2个、110千伏输变电工程9个和35千伏输变电工程14个。力争用三年时间，全面完成未改村的村级电网改造，实现县县建有220千伏变电站。结合新一轮农网改造工作，完善大圳灌区体制改革，努力争取大圳灌区电力公司上划。

　　加快扩大消费需求，促进三产繁荣活跃。坚持扩大内需。全年新建、改造"万村千乡"配送中心2个、农贸市场5个和农家店500个。支持物流业发展壮大，引导健身和文化等新型消费健康发展。抓好招商引资。突出工业、生物资源、新型能源、高新技术、旅游等重点领域，全面推行网上招商、邵商招商、产业招商，力争在对接央企投资和承接沿海产业转移等方面取得新的成绩。筹办好中博会、湖南省邵阳市（北京）投资推介会等重大招商活动。全年引进外资1.51亿美元、内资486亿元，分别增长10%、15%。发展外经外贸。扶持玉新药业、立得皮革、李文食品等出口龙头企业，培育出口新兴主体和主导产品。引导建筑、机械、农业水电等优势产业向东盟扩张。全年新批境外投资企业13家，实现国际劳务输出4200人。完成进出口总额4.5亿美元，增长15%。创新金融服务。支持资本、保险市场发展，争取1至2家股份制银行入邵设立分支机构。发挥城市建设融资平台作用，增强城建投集团融资能力。全年各金融机构贷款增长20%以上，力争维克液压发行上市，新增小额贷款公司5家、融资性担保公司4家和村镇银行2家以上。加快农村信用社改制为农村商业银行步伐。壮大旅游产业。以"文化旅游年"为主题，加大旅游宣传力度。完成崀山5A景区创建初评工作，确保白水洞申报国家级风景名胜区成功，启动蔡锷故居、紫薇博览园、莲荷生态公园国家等级景区的创建工作，加快湘窖生态酿酒园和湖南世博馆重建工程进度，完善崀山、南山、虎形山花瑶、云山、黄桑、 溪等景区景点规划和建设。竣工3家高星级旅游饭店，培育2家旅行社晋升高星级，建成邵阳市游客服务中心。争取全年接待游客超1400万人次，旅游总收入达到100亿元以上。

　　　2、努力促进各项事业全面进步

　　繁荣文化事业。推动文化大发展、大繁荣，加快建设文化强市。实施文化惠民工程，抓好"三馆一站"免费开放，继续做好"送戏下乡"和公益电影放映工作。提高广播电视覆盖质量。深化文化体制改革，完成公益性文化事业单位内部机制改革，推进经营性文化单位转企改制。加强基层文化建设，全面完成乡镇综合文化站建设工程。加快国家"文化信息资源共享工程"市县级中心和市博物馆建设。培育骨干文化企业，推动文化产业成为支柱产业。重视文物保护和文化遗产抢救。强化文化市场监管。办好第二届邵阳市艺术节。

　　优先发展教育。加大义务教育合格学校建设力度，继续实施农村中小学危房改造、初中校舍改造和特殊学校、幼儿园建设工程。加强学前教育的统筹管理，巩固提高义务教育，协调发展高中教育。全年义务教育完成率保持在98%以上。在全市范围基本普及高中阶段教育。加强职业教育，努力创建国家级示范性职业学校、国家级重点实训基地、国家级重点职业中专和省级示范性中等职业学校、省级示范性职教中心。支持邵阳学院和邵阳医专联合组建湖南经济学院，争取邵阳师范和武冈师范整合申办专科院校。

　　改善医卫条件。深化医药卫生体制改革，巩固基本药物制度成果。加强医疗卫生基础设施建设，完善医疗服务格局，加快市中心医院异地扩建工程、市中医院门诊综合楼、市一人民医院儿科综合楼建设步伐，建设市传染病医院、市肿瘤医院和市儿童医院。支持湘雅医院在江北建立邵阳分院，启动市卫生监督所建设项目，建设湘西南血液检测中心。加强传染病防控工作，有效控制手足口病等重大疫情。深入开展城乡居民健康教育和爱国卫生运动，保障公众健康和生命安全。

　　鼓励科技创新。支持高新技术产业发展，培育科技中小微型企业5至8家，新增高新技术企业3至5家，重点培育5家科技型示范企业，引进开发新产品50个。专利申请量超过1000件，专利授权达到600件。培育市级研发机构5个，创建省级工程技术研究中心1至2家，组建邵阳农业科学研究院。争取产学研合作签约项目50个，组建产学研战略联盟2至3个。

　　做好计生工作。加强基层基础工作，着力落实和完成人口计生利益导向政策；加强部门综合治理和流动人口服务管理，注重诚信计生建设，不断提高计生队伍依法行政能力和群众自治水平。支持绥宁、邵东、新邵和武冈四县市创建国家人口计生优质服务县。全市符合政策生育率稳步提升，出生性别比有所下降。

　　与此同时，加大体育基础设施建设力度，广泛开展全民健身运动。动工修建体育中心，申办2018年省运会。深入开展"双拥"活动。强化国家安全观念。支持人民解放军和武警部队建设，抓好国防动员、民兵预备役和人防事业，进一步落实军人优待抚恤政策，做好军队转业干部和复员退伍军人接收安置工作。加快市民兵综合训练基地和人防疏散基地建设步伐。健全企事业单位统计名录库，全面实施企业统计联网直报。加强工商、质监、市志、档案、贸促、移民、宗教管理、人事编制、外事侨务、邮政通讯、气象水文、防震减灾、市志档案、妇女儿童和无线电管理等工作。

　　3、努力创造人民群众幸福生活

　　办好实事工程。严格落实各项惠民政策。认真办好省市两级确定的实事工程，确保各项指标全面完成或超额完成目标任务。

　　增加社会就业。开展多层次、多形式的职业技能培训和转岗就业培训。实施"创业扶持计划"，提高困难企业下岗职工、高校毕业生、返乡农民工三大群体就业创业能力。健全服务、培训、维权三位一体的工作机制，有效组织劳务输出。推进就业困难人员就业援助工程，加强公益性岗位开发管理。扶持小微企业发展，充分吸纳社会就业。创建邵阳技师学院。争创省级创业型城市。全年完成培训10万人，新增城镇就业人员4.5万人。

　　完善社会保障。完善养老、医疗保险关系转移接续办法，开展医疗保险异地就医管理服务，做好医疗保险市级统筹，加快实现城镇居民养老保险全覆盖。开工建设邵阳社保大楼。全年实现城镇社保扩面10万人，基金征缴20亿元以上，发放保障资金40亿元以上。加强救灾救济、城乡低保和城乡医疗救助，进一步提高救助水平。加大残疾人事业发展力度。新建和改扩建敬老院16所，提高五保对象的生活供养标准。抓好苏区县申报争取工作。继续加强殡葬管理，整顿殡葬秩序。加快保障性住房建设，新建廉租房31.9万平方米、6382套，新建公租房18.5万平方米、3081套，改造棚户区29.3万平方米、6350户，发放租赁补贴3600万元。加大农村危房改造力度。依法照章发挥住房公积金在住房保障和公租房建设中的作用。

　　推进扶贫开发。抓住国家在武陵山片区率先开展区域发展与扶贫攻坚试点机遇，围绕稳定解决扶贫对象温饱、尽快实现脱贫致富两个目标，突出整村推进、产业开发和劳务培训三大重点，加快推进产业扶贫、基础扶贫、科教扶贫、生态扶贫和社会扶贫。依托中富油脂、瑞柏茶油等龙头企业，建设油茶、玉米等生产基地，帮助干旱贫瘠地区群众增收。全年争取财政扶贫资金2亿元、贴息贷款资金6亿元，直接帮扶11.7万人脱贫致富。

　　抓好安全生产。强化安全隐患排查整治，深化"打非治违"专项行动，确保不发生重大以上安全生产事故。争创全省安全生产先进单位。切实抓好防汛抗旱工作，严密防范山洪地质灾害。健全预警监测系统，保障人民群众饮食用药安全。

　　维护社会稳定。推进社会管理创新，逐步完善政府主导、社会协同、公众参与的社会管理格局。加强社会管理综合治理，深入开展"打黑除恶"专项行动和治安重点整治，严厉打击"两抢一盗"等各类违法犯罪活动。加大"六五"普法力度，建设法治邵阳。巩固完善和全面推广洞口县群众工作站的经验。有效防范和及时化解社会矛盾，切实维护好企业和群众的合法权益。重视网络舆情管理，畅通群众诉求渠道，着力解决信访中的突出问题。

　　保护生态环境。继续巩固退耕还林成果，全面推进石漠化综合治理。深入开展植树造林，完成衡邵和邵永高速公路绿色通道、资江风光带以及城边、路边、水边的绿化美化。推广清洁能源，完成户用沼气池1万口，建设大型沼气工程4处。推进节能、节地、节水、节材工作。划定永久性基本农田，加大耕地垦复力度，保持耕地总量动态平衡。加强城市环境综合治理，推进农村环境污染综合整治，整顿规范矿产资源开发秩序。开展龙须塘化工区废水深度治理工程，抓紧对7条中小河流进行治理。集中整治污染隐患，抓好城区14家重污染企业的取缔关闭、23家污水直排企业的停产整顿、13家污染物超标排放企业的限期治理，确保完成年度污染减排任务。

　　各位代表，在今年的工作中，我们将始终注重加强政府自身建设。一是严格依法照章办事。自觉接受人大及其常委会的法律监督、工作监督和政协的民主监督，认真办理人大代表议案、建议和政协提案，经常向人大报告和向政协通报协商重大事项，全面落实人大的决议决定和审议意见，重视采纳政协的调研成果和意见建议。广泛听取民主党派、工商联、无党派人士和各人民团体的意见，主动接受社会公众和新闻舆论监督。二是创优经济发展环境。充分发挥监察和审计的职能作用，严格行政权力运行监督。认真落实《湖南省政府服务规定》，继续扩大政务公开，不断改进政府服务。三是保持清正廉洁形象。全体政府工作人员尤其是各级领导干部不仅要作干事创业的模范，而且要当清正廉洁的表率。

　　各位代表，2012年是本届政府任期最后一年，做好今年政府工作意义非常重大。让我们在中共邵阳市委的正确领导下，为完成或超额完成今年各项工作任务、实现本届政府向全市人民承诺的各项发展目标而努力奋斗！
